# Peptide-functionalized iron oxide magnetic nanoparticle for gold mining

**DOI:** 10.1007/s11051-017-3752-7

**Published:** 2017-02-17

**Authors:** Wei-Zheng Shen, Sibel Cetinel, Kumakshi Sharma, Elham Rafie Borujeny, Carlo Montemagno

**Affiliations:** 1 Ingenuity Lab, 1-070C, 11421 Saskatchewan Drive NW, T6G 2M9, Edmonton, AB Canada; 2 Departement of Chemical and Materials Engineering, University of Alberta, T6G 2V4, Edmonton, AB Canada; 3 National Institute of Nanotechnology, 11421 Saskatchewan Drive NW, T6G 2M9, Edmonton, AB Canada

**Keywords:** Gold-binding peptide, Magnetic nanoparticles, Surface functionalization, Gold mining, Nanobiotechnology

## Abstract

Here, we present our work on preparing a novel nanomaterial composed of inorganic binding peptides and magnetic nanoparticles for inorganic mining. Two previously selected and well-characterized gold-binding peptides from cell surface display, AuBP1 and AuBP2, were exploited. This nanomaterial (AuBP-MNP) was designed to fulfill the following two significant functions: the surface conjugated gold-binding peptide will recognize and selectively bind to gold, while the magnetic nano-sized core will respond and migrate according to the applied external magnetic field. This will allow the smart nanomaterial to mine an individual material (gold) from a pool of mixture, without excessive solvent extraction, filtration, and concentration steps. The working efficiency of AuBP-MNP was determined by showing a dramatic reduction of gold nanoparticle colloid concentration, monitored by spectroscopy. The binding kinetics of AuBP-MNP onto the gold surface was determined using surface plasmon resonance (SPR) spectroscopy, which exhibits around 100 times higher binding kinetics than peptides alone. The binding capacity of AuBP-MNP was demonstrated by a bench-top mining test with gold microparticles.

**Electronic supplementary material:**

The online version of this article (doi:10.1007/s11051-017-3752-7) contains supplementary material, which is available to authorized users.

**Figure:**
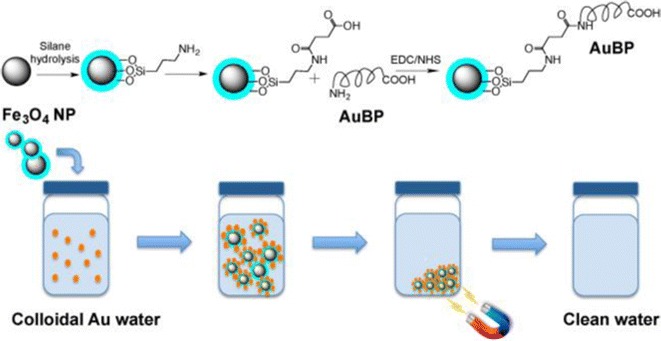
Graphical abstract

## Introduction

Tailings are the by-products left over from mining and extracting resources. Depending on the resource being mined, processing technology used and geology at the mine site, the type, amount, and properties of mine waste found in different tailings vary. Much of this waste contains very precious minerals, such as noble metals, semiconductors, and rare earth elements. However, mixed with sand, silt, clay, chemicals, and water, these valuable minerals are generally difficult to recover and are usually deposited in the form of a water-based slurry into tailing ponds. These leftover minerals represent billion of dollars in loss. Moreover, the seeping of the mine water containing dissolved heavy minerals has been known to contaminate surface and groundwater, adversely affect aquatic life, and cause severe health problems (Frank et al. 2014; Nuss and Eckelman 2014). A novel emerging technology is needed to develop an environmentally sustainable and cost-effective way to extract these valuable materials from tailings without excessive solvent extraction, filtration, and concentration steps.

Research into the use of magnetic iron oxide nanoparticles as tools for heavy metal remediation has increased in recent times because their high surface area enables enhanced heavy metal uptake (Carlos et al. 2013), their strong magnetic response grants easy separation in a magnetic field, and their low toxicity mitigates the environmental impact. An important aspect in the fabrication of magnetic iron oxide nanoparticles for their use in heavy metal recovery is the attachment of functional groups onto the surface of the nanoparticles. The use of functional groups for surface modification is particularly important, as it provides the nanoparticles with specific material recognition and binding abilities.

Combinatorial biology tools have enabled researchers to select peptides for various inorganic materials through molecular recognition and binding (Sarikaya et al. 2003; Tamerler and Sarikaya 2007; Tamerler and Sarikaya 2009; Tamerler et al. 2010). Despite the fact that the mechanism of how peptides interact with other/various materials has not yet been fully understood, quantitative binding experiments and modeling give some clues of how this might be possible. It is generally believed that the recognition is caused by the synergy of polarity, hydrophobicity, charge, and matched conformation of peptides and inorganic surface. Therefore, this kind of interaction could easily break by the change of the environment, such as the pH and ionic strength of the buffer used, and competitive binding. Up to now, more than 20 different sets of peptides have been biocombinationally identified and are capable of recognizing the surfaces of a variety of inorganic materials (Whaley et al. 2000). Among them, gold-binding peptides were the first group of peptides to be selected and characterized (So et al. 2009a; So et al. 2009b). Because of the intensive study in the gold-peptide interaction, particularly in terms of binding kinetics and free energy, researchers can understand, engineer, and control peptide-material interactions and exploit these to tailor novel materials and systems for practical applications (Hnilova et al. 2008; Hattori et al. 2012; Niide et al. 2013; Seker et al. 2014).

This paper presents work on preparing a novel nanomaterial composed of gold-binding peptides conjugated on the surface of shell-protected magnetic nanoparticles. These materials have a magnetic core that facilitates their recovery by responding and migrating according to the applied external magnetic field. The shell provides stability, protection from oxidation, and a surface to which the gold-binding peptide can conjugate to. As a first proof of concept for our envisaged strategy, we demonstrate herein that the gold-binding, peptide-functionalized, magnetic nanoparticles have a precisely defined chemical composition, size, shape, and functionality. In particular, they lead to efficient extraction of gold from solution.

## Experimental section

### Materials and methods

AuBP1 and AuBP2 peptides were obtained from AAPPTEC, LLC. Iron chloride (FeCl_3_⋅5H_2_O), sodium hydroxide (NaOH), ethylene glycol (EG), 3-aminopropyltriethoxysilane (APTES), succinic anhydride, dichloromethane (DCM), 1-ethyl-3-(3-dimethylaminopropyl) carbodiimide hydrochloride (EDC), N-hydroxysulfosuccinimide **(**sulfo-NHS), 2-(*N*-morpholino)ethanesulfonic acid (MES), PBS buffer, and gold microparticles were purchased from Sigma-Aldrich (Canada). All chemicals were used as received with no further purification.

SEM and TEM images of nanoparticles were taken using a Hitachi S-4800 field emission electron microscope and a JEOL TEM-2200FS transmission electron microscope. Samples for SEM and TEM were prepared by dripping 5 μL of dilute sample solution onto a carbon-coated copper grid, blotting excess liquid with filter paper after 1 min, and drying at room temperature. X-ray powder diffraction (XRD) spectra were recorded on a XRD-6000 diffractometer in the 2*θ* range of 15–80° with Cu-Kα radiation (*λ* = 0.154060 nm) and a scanning rate of 0.05 deg s^−1^. X-ray photoelectron spectra (XPS) were acquired on a Kratos AXIS 165 electron spectrometer with 150-W monochromatized A1-Kα radiation (1486.6 eV), where all peaks were referred to the signature C1s peak for adventitious carbon at 284.8 eV. Fourier transform IR (FTIR) spectra were recorded on a Nicolet 6700 Fourier transform infrared spectrometer in the range of 400–4000 cm^−1^. Field-dependent magnetization was measured on the superconducting quantum interference device (SQUID, Quantum Design, MPMS-XL-7T) magnetometer at 300 K. Hydrodynamic diameters and zeta potential were obtained using a Malvern Nano-Z instrument. UV-Vis spectra were collected on Agilent 8453 UV-Vis spectrophotometer. Thermogravimetric analysis was conducted using a Thermal Analysis Instrument TGA Q50 (TA Instruments) apparatus under a flow of nitrogen to study the thermal stability of the nanoparticles and their organic components. Surface plasmon resonance (SPR) measurements were accomplished in a Biacore-X instrument (GE Healthcare, Biacore Life Sciences). The concentration of the Fe_3_O_4_ NPs and the amount of gold adsorbed were determined by using a PerkinElmer Elan6000 quadrupole ICP-MS, combined with a UP213 (Merchantek) laser ablation system.

### Synthesis of Fe_3_O_4_ nanoparticles

Highly uniform quasi-spherical iron oxide magnetic nanoparticles (MNPs) (~70 nm) were synthesized by using a hydrothermal reaction with a slight modification (Ge et al. 2009). Briefly, 0.5 g of FeCl_3_ and 0.2 g of NaOH were dissolved in 15 mL of EG at 50 °C to obtain an orange-yellow suspension. Then, the suspension was transferred into a 50-mL PPL chamber in a stainless steel autoclave reactor. The autoclave reactor was sealed and heated up to 240 °C for 5 h. The Fe_3_O_4_ NPs formed as a black precipitate in the polyphenyl chamber. The collected Fe_3_O_4_ NPs were magnetically separated from the reaction mixture and washed thoroughly with ethanol five times and finally freeze-dried, yielding 180 mg of Fe_3_O_4_ NPs powder.

### Amine/carboxylate functionalization of Fe_3_O_4_ nanoparticles (Fe_3_O_4_@APTES, Fe_3_O_4_@OPTBA)

Amine functionalization of Fe_3_O_4_ nanoparticles was achieved via APTES hydrolysis and condensation. In a typical procedure, 0.5 mL of APTES was added slowly to the suspension of 30 mg of Fe_3_O_4_ nanoparticles, prepared in a mixture of 200 mL isopropanol, 2 mL H_2_O, and 1 mL NH_3_⋅H_2_O under sonication for 30 min. Then, the mixture was kept in the ultrasonic bath at 50 °C for 3 h. The resulting Fe_3_O_4_@APTES were collected by magnetic separation and washed with isopropanol five times to remove the unreacted APTES and dried into powder in the freeze dryer.

Carboxylate functionalization of Fe_3_O_4_ nanoparticles was achieved via a two-step reaction, namely, APTES was converted into 4-oxo-4-(3-(triethoxysilyl)propylamino)butanoic acid (OTPBA) via succinic anhydride conjugation, and then, OTPBA was introduced to the nanoparticle surface via silane hydrolysis and condensation. In a typical procedure, 1.0 g succinic anhydride was dissolved in dry dichloromethane (100 mL) at 0 °C with an ice bath and 2.4 mL of APTES was added slowly into the solution within 1 h. The reaction was kept stirred at room temperature for another 5 h. Afterwards, the solvent was removed by rotovap and it left ~2 mL of a light yellow, oily liquid. ESI-MS (*m*/*z*) found (calcd) 320.33 (320.16), [M-H]^−^ (C13H27NO6Si).

Of the OTPBA, 0.5 mL was added slowly to the suspension of 30 mg Fe_3_O_4_ nanoparticles, prepared in a mixture of 200 mL isopropanol, 2 mL H_2_O, and 1 mL NH_3_⋅H_2_O under 30-min sonication. The mixture was kept in the ultrasonic bath at 50 °C for 3 h. The resulting Fe_3_O_4_@OTPBA were collected by magnetic separation, washed with isopropanol five times, and dried into powder in the freeze dryer.

### Quantification of amine/carboxyl group

The functionalization of NH_2_/COOH was quantified by either colorimetric assay of amine density, utilizing 4-nitrobenzoaldehyde (4-NBA) (Xiang et al. 2012), or Ni^2+^ titration together with pyrocatechol violet (PV) for carboxyl density (Hennig et al. 2011).

#### Amine quantification

Briefly, 5 mg of Fe_3_O_4_@APTES were placed in a 1.5-mL centrifuge tube and washed four times with 1 mL of coupling solution (0.8% (*v*/*v*) glacial acetic acid in dry methanol). Of the 4-NBA solution (7 mg in 10 mL of coupling solution), 1 mL was added to the particles and the suspension was allowed to react for 3 h with gentle end-over-end rotation. After removal of the supernatant and being washed four times with 1 mL of coupling solution, 1 mL of hydrolysis solution (75 mL of H_2_O, 75 mL of methanol, and 0.2 mL of glacial acetic acid) was added to the particles, and the tube was shaken for another hour. The supernatant was then removed from the particles with a magnetic separator, and its absorbance was recorded at 282 nm. The amount of 4-NBA in the hydrolysis solution was calculated by interpolation using a calibration curve constructed from a range of standard solutions of 4-NBA, each prepared separately.

#### Carboxyl quantification

All the experiments were carried out in 10 mM of HEPES buffer (pH = 7.5). First, the stock solution of Fe_3_O_4_@OPTBA was prepared by dispersing 5 mg of NPs in 1 mL of buffer. Then, varying amounts of the Fe_3_O_4_@OPTBA stock solutions (100, 200, 300, and 400 μL) were incubated with 1.2 mM Ni^2+^ for ~2 min in a total volume of 1000 μL of the buffer. After centrifugation for 15 min at 13,000 rpm, 500 μL of the supernatant was diluted into 1000 μL with 498 μL of HEPES buffer and 2 μL of a freshly prepared PV stock solution (20 mM). Absorption spectra were recorded immediately after PV addition and mixing. The absorbance at 650 nm was plotted against the particle stock solution volume. Linear fitting of the initial linear decrease of this titration plot gave the slope of the fitted line *a*, the *y* intercept *b*, and the number of surface carboxyl groups obtained by Eq. 1. This procedure was repeated three times to afford the amount of extracted Ni^2+^.

1 $$ \mathrm{Surface}\ \mathrm{carboxy}\ \mathrm{groups}\ \left(\frac{\upmu \mathrm{mol}}{\mathrm{g}}\right)=\frac{n\left[{\mathrm{M}}^{2+}\right] Va}{w\left({A}_{PV}- b\right)} $$

where *V* is the volume, [M^2+^] is the metal ion concentration during particle/M^2+^ incubation, *A*_*PV*_ is the absorbance of PV in the absence of M^2+^, *w* is the mass concentration (in mg/mL) of the particle stock solution, and *n* is a stoichiometry factor indicating the number of surface carboxyl groups per metal cation (*n* = 2.65 for Ni^2+^).

#### Coupling reaction of AuBPs and Fe_3_O_4_@OTPBA NPs

Gold-binding peptides (AuBP1 and AuBP2) were introduced to Fe_3_O_4_@OTPBA NPs via the EDC/NHS-mediated coupling reaction. Of the Fe_3_O_4_@OTPBA NPs, 30 mg was dispersed in 15 mL of MES buffer (pH = 6) under a 5-min sonication. Then, the carboxylate groups were activated by addition of EDC/sulfo-NHS (final concentration 3.6 and 7.2 mM) and were shaken for 2 h. The excess EDC/sulfo-NHS was washed away by magnetic separation and re-dispersion in Milli-Q water five times, then dried in the freeze dryer. Of the carboxylate-activated Fe_3_O_4_@OTPBA@sulfo-NHS NPs, 30 mg was dispersed 15 mL of 1 mg/mL AuBP in a PBS buffer, and the reaction was allowed to complete overnight. The non-reacted activated carboxylic acid groups were quenched by incubating the NPs in 50 mM ethanolamine in PBS for 1 h at room temperature. The resulting Fe_3_O_4_@OTPBA@AuBP NPs were washed in PBS buffer five times under magnetic separation and re-dispersion.

#### Quantification of the covalently conjugated AuBP

To determine the coating density of AuBP on the Fe_3_O_4_@OTPBA@AuBP NPs, 1 mg/mL of AuBP1 and AuBP2 was prepared in PBS and the absorptions at 280 nm (aromatic amino acids) were recorded, respectively. Of the freeze-dried Fe_3_O_4_@OTPBA@NHS NPs, 5 mg was transferred into 1.5 mL of the 1 mg/mL AuBP solution for an overnight reaction. The reaction was stopped the next day by separating the MNPs from the solution via centrifugation. The absorption of the supernatant at 280 nm was recorded in comparison with that of the peptide solution before the reaction. The reaction was repeated three times for each peptide, with three repeats of non-activated Fe_3_O_4_@OTPBA as the negative control.

### Gold adsorption test

#### Gold nanoparticle-binding test in water

A THPC gold nanoparticle colloid solution was prepared by following the method reported earlier (Duff et al. 1993). Of the NP, AuBP1-NP, and AuBP2-NP, 1 mg was added separately into 2 mL of THPC gold nanoparticle colloid solution. Vortex was applied to ensure the interaction of the nanoparticles. After 5 min, a magnet was applied to remove the magnetic nanoparticle from the solution. The UV-Vis spectra were recorded for the resulting supernatants.

#### SPR measurement

Experiments were performed with Au-coated SPR sensor chips and at a temperature of 22 °C. Before the experiments, 1 mg/mL stock NP solutions (1.96 × 10^3^ pM for NP, 1.87 × 10^3^ pM for AuBP1-NP and AuBP2-NP) were prepared in a phosphate buffer (1× PBS). PBS was pumped into the system with a 50-μL/min flow rate until the stable baseline signal was established. Then, 100 μL of the NP solution was injected and the adsorption was monitored. Subsequently, in order to observe desorption, PBS was pumped in until the new baseline was established. At least three different NP concentrations were tested in order to extract kinetic and thermodynamic properties of the system using the Langmuir adsorption model.

Raw kinetic data from SPR tests were fitted by least squares regression to a 1:1-L adsorption model per

$$ \frac{ d S(t)}{ d t}={k}_a\ \left({S}_{\max }- S(t)\right)\  C-{k}_d\  S(t) $$

where *S*(*t*) is the resonance signal response corresponding to occupied sites at time *t*, *C* is the bulk concentration of the nanoparticle, and *S*_max_ is the response for the case that all the binding sites on the sensor are occupied by nanoparticles.

Therefore, time evolution of the resonance signal is obtained from the solution of the aforementioned differential equation, subject to this initial condition that at *t* = *t*_0_, *S* = 0 per

$$ S\left( t-{t}_0\right)={S}_{\max}\frac{k_a C}{k_a C+{k}_d}\ \left(1-{e}^{-\left({k}_a C+{k}_d\right)\left( t-{t}_0\right)}\right) $$

where ${S}_{\max}\frac{k_a C}{k_a C+{k}_d}={S}_{\mathrm{eq}}$ is the response at equilibrium. Assuming a linear relationship between the raw response signal (*S*) and surface coverage (*θ*), it results in the following expression for the surface coverage:

$$ \theta \left( t-{t}_0\right)={\theta}_{\mathrm{eq}}\left[1- \exp \left(-{k}_{obs}\left( t-{t}_0\right)\right)\right] $$

where *θ*_eq_ is the equilibrium surface coverage and *k*_obs_ = *k*_*a*_ *C* + *k*_*d*_.

The adsorption (*k*_*a*_) and desorption (*k*_*d*_) rates were calculated by determining *k*_obs_ at several concentrations. From these kinetic constants, the equilibrium constant (*K*_eq_) and Gibbs free energy change of adsorption (∆*G*) were also calculated.

Gold-mining test in water: To determine the ability of AuBP-NP to remove Au^0^ from solution, gold microparticles (40 μm) were utilized. A light red colored gold suspension was prepared by dispersing 20 mg of gold powder in 5 mL of Milli-Q water, and the pH was recorded to be 6.8. Then, 1 mg of dried Fe_3_O_4_@OPTBA@AuBP powder was added into the gold suspension, followed by 2-min, end-to-end shaking. A neodymium magnet was applied to the resultant solution for another 2 min. Then, the supernatant was separated from the magnet-attracted solid. Freshly prepared aqua regia was added into both the solution and the solid, forming 10 mL of a final yellow solution. The concentrations of Au(III) and Fe(III) were determined by ICP-AES analysis using three runs and three samples.

## Results and discussion

### Preparation of Fe_3_O_4_@OPTBA@AuBP NPs

**Scheme 1:**
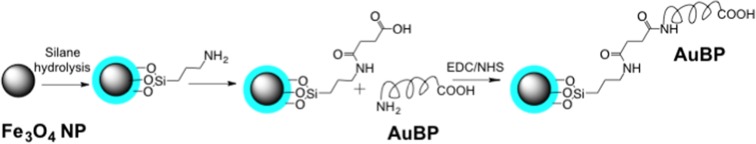
Preparation of Fe_3_O_4_@OPTBA@AuBP NPs

As shown in Scheme 1, gold-binding, peptide-functionalized, magnetic nanoparticles were prepared using a three-step protocol under aqueous reaction conditions at room temperature. First, iron oxide MNPs were synthesized by using a hydrothermal reaction with slight modification (Ge et al. 2009). Second, amine or carboxylate groups were introduced onto the surface of the magnetic nanoparticle via silane hydrolysis and condensation (Bruce and Sen 2005). Third, gold-binding peptides (AuBP1 and AuBP2) were introduced to the functionalized silane via a EDC/NHS-mediated coupling reaction.

The size of the NPs could be precisely tuned by adjusting the concentration of the precursor and reaction time. For example, using a lower concentration of the precursor and the same reaction time, smaller NPs with uniform sizes of 40, 50, and 70 nm could be obtained; using a higher concentration of precursor and a longer reaction time, larger nanoclusters with uniform sizes of 150, 180, and 250 nm could be reachable (SEM pictures of nanoclusters are shown in Fig. S1).

The gold-binding peptide (AuBP1 and AuBP2) was loaded onto Fe_3_O_4_@OPTBA in two steps. First, Fe_3_O_4_@OPTBA was activated by EDC/sulfo-NHS in a MES buffer (pH = 6.0) to form a stable Fe_3_O_4_@OPTBA@sulfo-NHS salt formation. Second, the peptide was tethered to Fe_3_O_4_@OPTBA through an amide bond formation in PBS buffer (pH = 7.4). Although in theory the formation of an amide bond is favorable in a basic condition, we observed a non-preferable peptide conjugation in 0.1 M of NaHCO_3_ buffer (pH = 10.0), most likely due to a base-catalyzed silane hydrolysis.

Zeta-potential measurement results show changes in surface charge during each step of the coating. Initially, Fe_3_O_4_@OPTBA had a typical, negative zeta potential −37.0 ± 2.90 mV at pH = 6.0, indicating a very stable, negatively charged colloid solution. After EDC/NHS activation, the total charge of the nanoparticles decreased slightly to −23.7 ± 1.86 mV. Table 1 represents the detail information of AuBP1 and AuBP2. They are both highly positively charged peptides. Therefore, both Fe_3_O_4_@OPTBA@AuBP1 and Fe_3_O_4_@OPTBA@AuBP2 became less negatively charged, −8.77 ± 0.69 and −5.21 ± 0.12 mV at pH = 6.0, respectively.

**Table 1:** Information of peptides AuBP1 and AuBP2

	AuBP1	AuBP2
Sequences	WAGAKRLVLRRE	WALRRSIRRQSY
*M*_*w*_	1454.7	1591.8
Theoretical pI	11.71	12.00
Charge	1 negatively charged; 4 positively charged amino acids	0 negatively charged; 4 positively charged amino acids
Absorbance (280 nm)	*ε* = 5500	*ε* = 6990
GARVY	−0.567	−1.267

### Characterization of Fe_3_O_4_@OPTBA@AuBP NPs

**Fig. 1:**
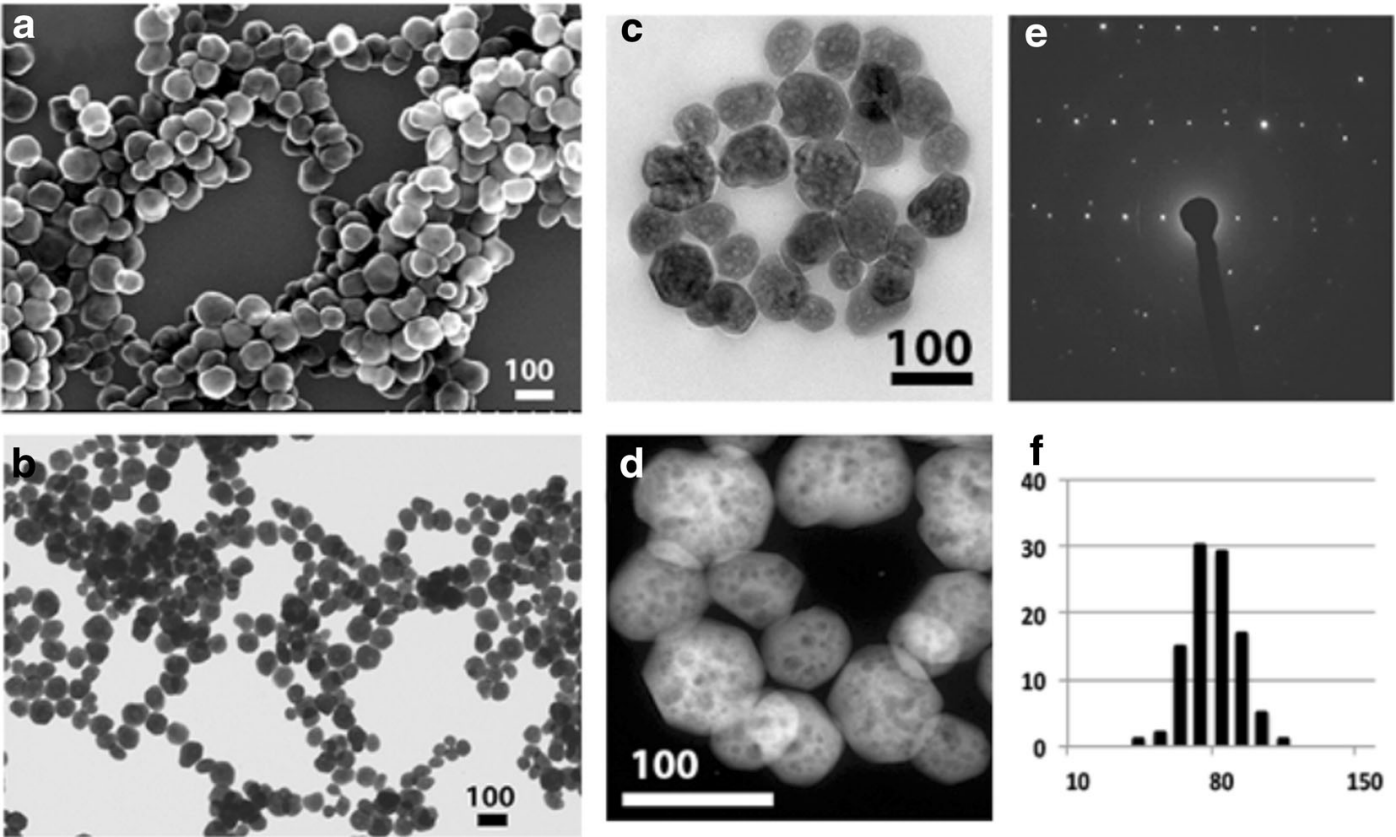
Comprehensive electron microscopic characterization of Fe_3_O_4_ NPs, including SEM (**a**), TEM (**b**), HR-TEM (**c**), HADDF (**d**), SAED (**e**), and size distribution (100 random pickup nanoparticles by ImageJ software)

A comprehensive electron microscopic characterization, including SEM, high-resolution TEM (HR-TEM), high-angle annular dark field (HADDF), and selected area electron diffractions (SAEDs) of the Fe_3_O_4_ NPs are shown in Fig. 1. The SEM (Fig. 1a) and TEM (Fig. 1b) images revealed that the as-prepared iron oxide nanoparticles had a quasi-spherical shape with fairly narrow size distribution at 70 ± 5 nm (Fig. 1f). The size distribution shown in Fig. 1f was obtained by measuring 100 randomly selected particles. HR-TEM (Fig. 1c) and HADDF (Fig. 1d) detected that there were bubble-shaped hollow structures with an average size of ~5 nm embedded in each NP. These bubbles might generate due to the high pressure and high temperature required for the formation of nanoparticles.

SAED (Fig. 1e) showed that these NPs were also nanocrystals with very regular diffraction patterns. XRD analysis revealed that the sites and intensity of the diffraction peaks were consistent with the standard pattern for ICSD collection code 77588 magnetite (Fe_3_O_4_) synthetic. The sample shows very broad peaks, indicating the ultra-fine nature and small crystallite size of the particles.

**Fig. 2:**
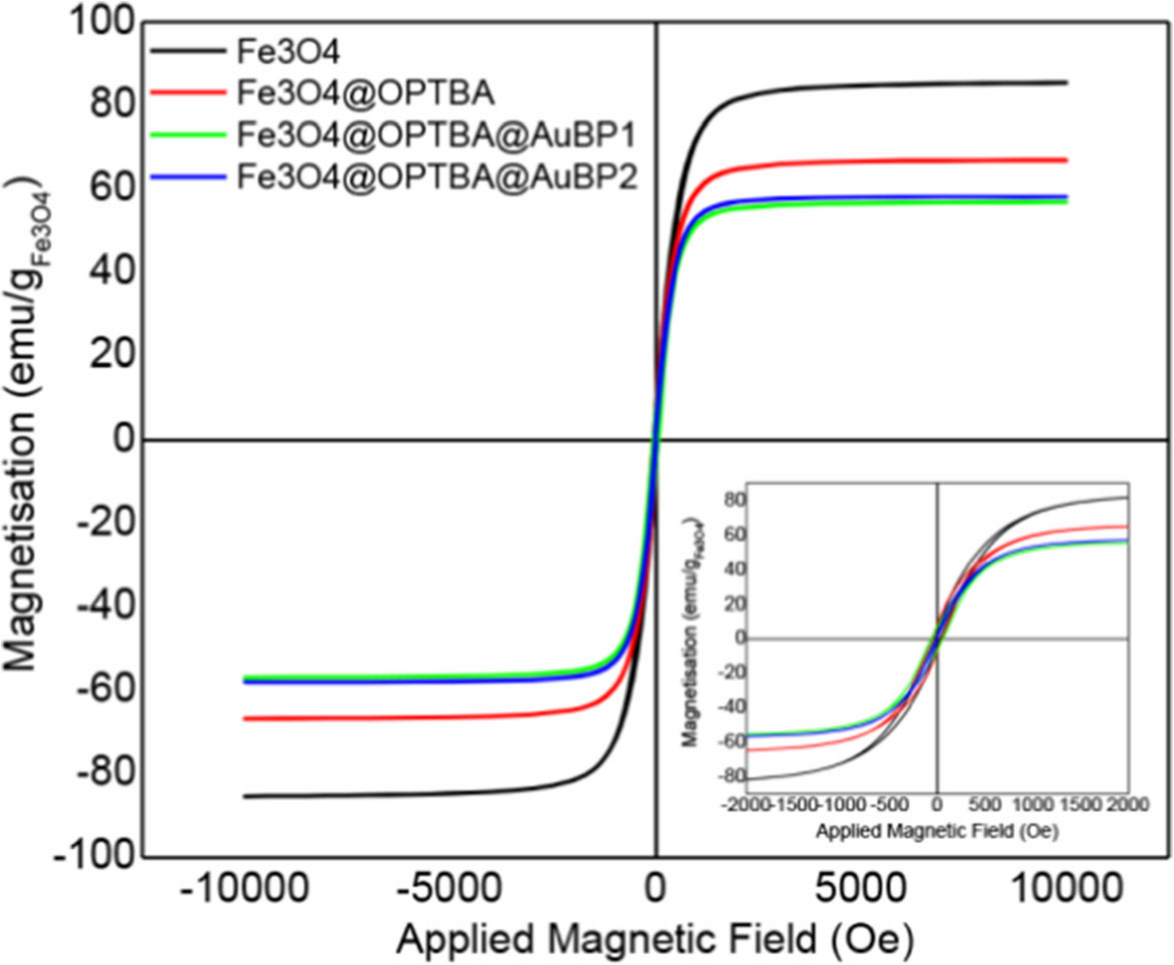
Field-dependent magnetization curves of Fe_3_O_4_, Fe_3_O_4_@OPTBA, Fe_3_O_4_@OPTBA@AuBP1, and Fe_3_O_4_@OPTBA@AuBP2 NPs at 300 K. *Insert* shows the magnification of magnetic hysteresis from −2000 to 2000 Oe at 300 K

The magnetic properties of Fe_3_O_4_, Fe_3_O_4_@OPTBA, Fe_3_O_4_@OPTBA@AuBP1, and Fe_3_O_4_@OPTBA@AuBP2 NPs were studied by applying an external magnetic field and recording their responding magnetizations at room temperature. The hysteresis loops are presented in Fig. 2. The initial Fe_3_O_4_ NPs had a saturated magnetization of 86 emu/g, only slightly lower than the bulk iron oxide material (92 emu/g). With the silane coating, the saturation magnetization of Fe_3_O_4_@OPTBA (67 emu/g) decreased slightly, as expected. Even after peptide functionalization, the Fe_3_O_4_@OPTBA@AuBP still had a strong magnetic response, with a value of 58 emu/g for AuBP2 and 56 emu/g for AuBP1. (The magnetization value was calculated according to the mass of iron oxide.) This reduction of the magnetization indirectly confirms the success of each functionalization step. The remanence and coercivity of all four NPs were close to 0 (inset in Fig. 2), indicating that these NPs do not retain magnetic moment when the external magnetic field is 0. This is the magnetic property that we would like to obtain, namely, that it has a strong magnetic response even when a small external magnetic field is applied but has 0 magnetic remanence, so that it is well dispersed in solution when the external magnetic field is removed.

**Fig. 3:**
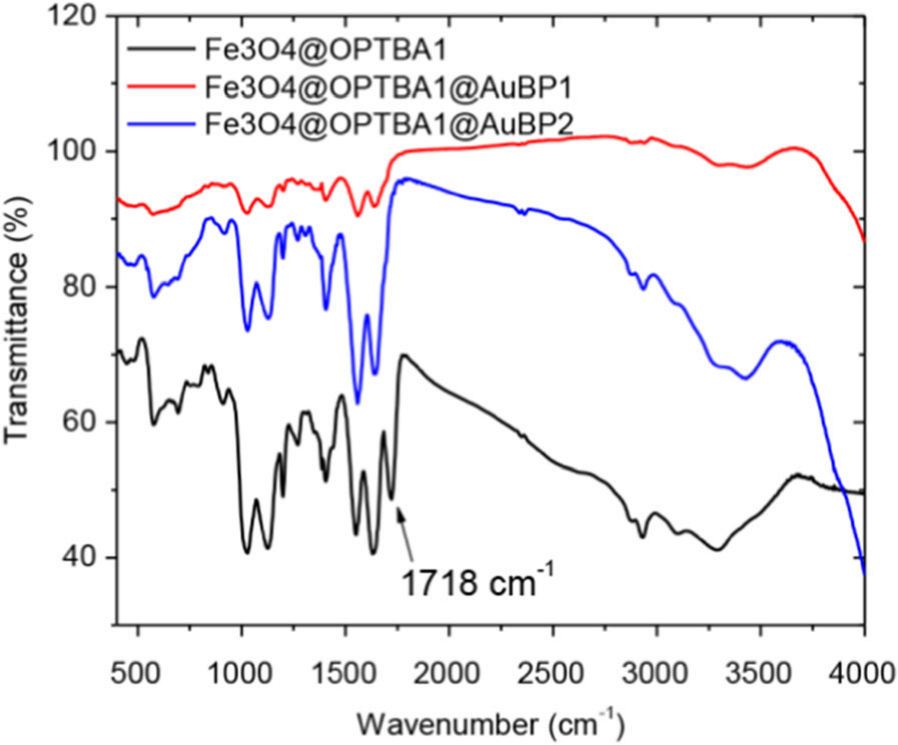
FTIR of Fe_3_O_4_@OPTBA, Fe_3_O_4_@OPTBA@AuBP1, and Fe_3_O_4_@OPTBA@AuBP2 NPs. Attributions (cm^−1^) 3293, *ν*_O–H_; 3199, *ν*_N–H_; 2931, *ν*_CH_; 1719, *ν*_C = O carboxylic acid_; 1643, *ν*_C = O amide_; 1553, *δ*_N–H_; 1402, *δ*_C–H_; 1196 *δ*_C–N_

Figure 3 shows the IR spectra of Fe_3_O_4_@OPTBA, Fe_3_O_4_@OPTBA@AuBP1, and Fe_3_O_4_@OPTBA@AuBP2. The strong IR band at 580 cm^−1^ is characteristic of the Fe-O vibrations related to the magnetite core, and the weak bands at 910 and 1030 cm^−1^ correspond to Si–OH and Si–O–Si or Si–O–Fe stretching vibrations of the silica shell. The strong IR bands at 1553 and 1633 cm^−1^ are characteristic of the amide bond for all three spectra. It should be noted that the peptide OPTBA also has an amide bond in its structure due to the conjugation between the amine and succinic anhydride. Therefore, the presence of an amide bond is not a good indication of peptide conjugation. Luckily, the IR spectra demonstrate a clear distinction between Fe_3_O_4_@OPTBA and Fe_3_O_4_@OPTBA@AuBP particles through the strong band at 1718 cm^−1^, corresponding to the C = O stretch vibration of the carboxylic acid. The disappearance of this band after the peptide conjugation demonstrates covalent binding of the peptide to the carboxyl groups of Fe_3_O_4_@OPTBA.

**Fig. 4:**
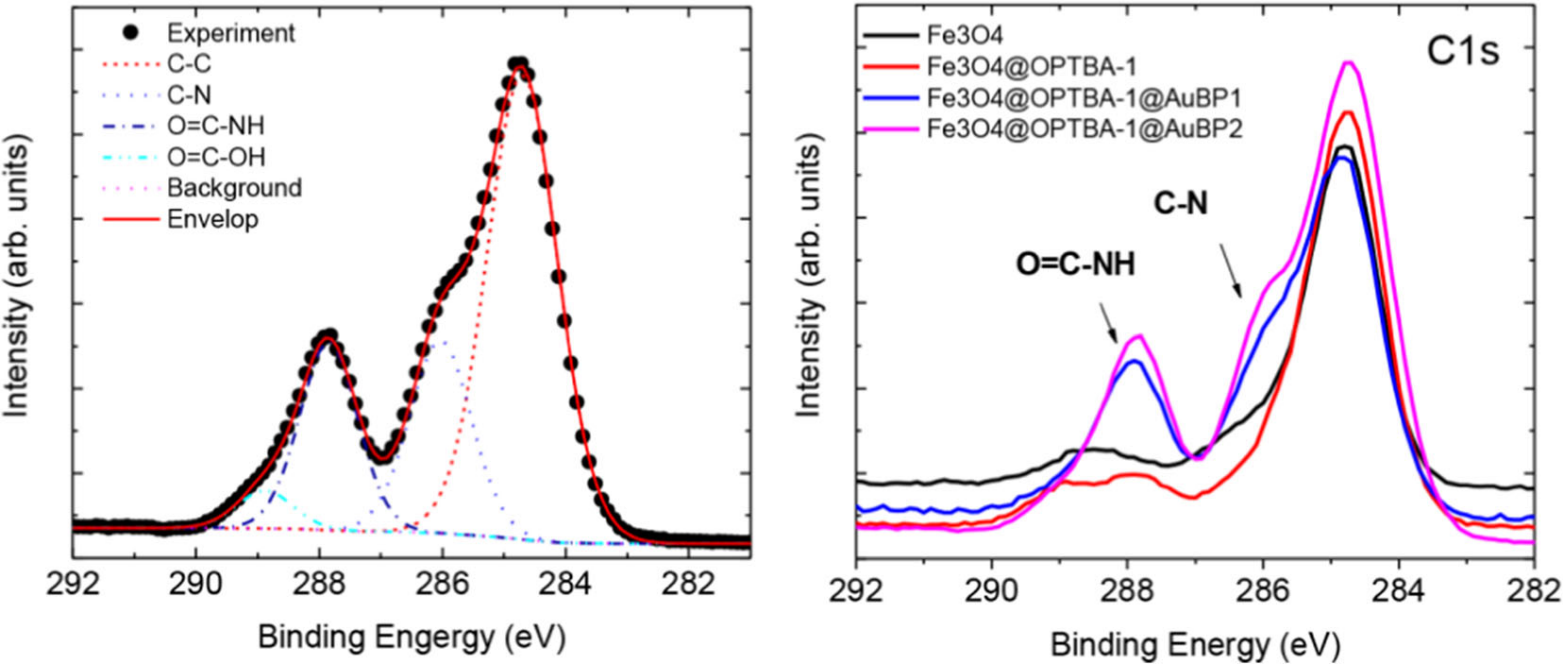
C1s high-resolution scan XPS spectra of Fe_3_O_4_, Fe_3_O_4_@OPTBA, Fe_3_O_4_@OPTBA@AuBP1, and Fe_3_O_4_@OPTBA@AuBP2 NPs (*right*), with respective deconvolutions for Fe_3_O_4_@OPTBA@AuBP2 (*left*)

To further elucidate the extent of the conjugation of NPs with peptides, a XPS study has been carried out. Figure 4 shows the high-resolution scans of XPS spectra of C1s in Fe_3_O_4_, Fe_3_O_4_@OPTBA, Fe_3_O_4_@OPTBA@AuBP1, and Fe_3_O_4_@OPTBA@AuBP2 NPs. On the left, the deconvolution of the C1s levels gives four peaks for all the samples, which are in accordance with most of the literature data. The peak at 285 eV corresponds to a carbon atom bound only to other carbon atoms and/or a hydrogen bond (C–C or C–H). The peak at 286.7–286.8 eV corresponds to a carbon bound to a single non-carbonyl atom (C-N). The peak at 288.2–288.3 eV represents a carbon atom bound to carbonyl oxygen and a single non-carbonyl atom (O = C–NH). The peak at 289.3–289.5 eV represents a carbon atom linked to one carbonyl oxygen and one non-carbonyl oxygen (O = C–OH). The relative intensity of each of the C1s level peaks was also represented in Fig. 4, on the right. Most importantly, peptide-functionalized NPs show much higher C–N and O = C–NH peaks, clearly indicating the success of peptide conjugation on the nanoparticle surface. In the comparison of Fe_3_O_4_ and Fe_3_O_4_@OPTBA, a higher C–C peak could be observed in Fe_3_O_4_@OPTBA, which indicates the increase of carbon atoms through the grafting of OPTBA. Even though the measurement is representing localized information and may not be suitable for an accurate quantification of the peptide on the nanoparticle surface, the relative intensity of AuBP1 and AuBP2 suggests that the reaction is preferential to AuBP2.

**Fig. 5:**
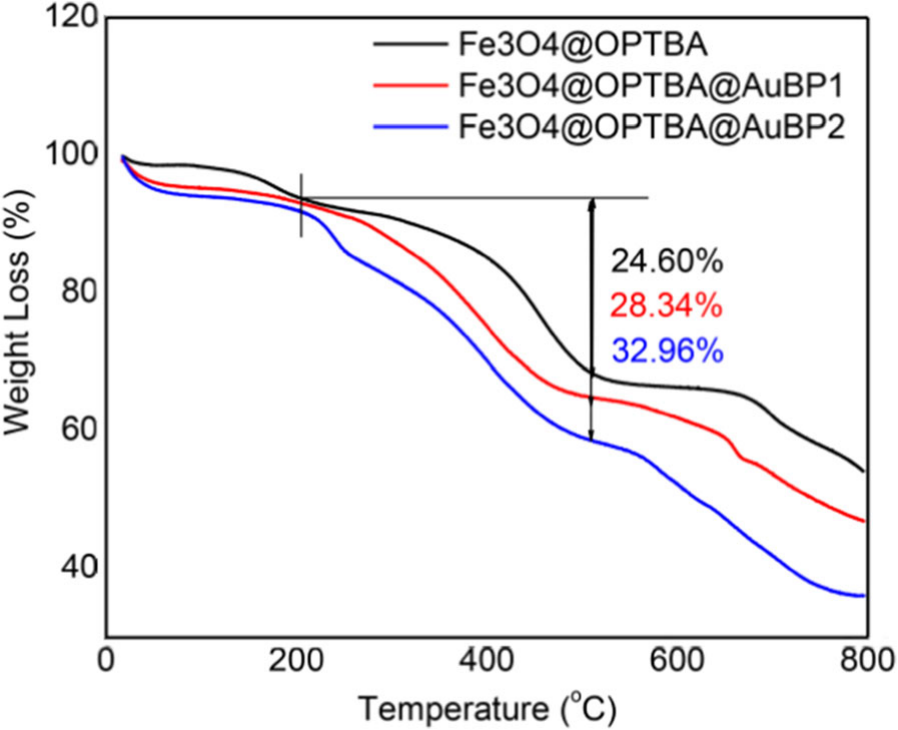
Thermogravimetric analysis of Fe_3_O_4_@OPTBA, Fe_3_O_4_@OPTBA@AuBP1, and Fe_3_O_4_@OPTBA@AuBP2

Thermogravimetric analyses were conducted to investigate the thermal degradation behavior of the obtained functionalized magnetic nanoparticles. Figure 5 shows the representative thermogram analysis curves for Fe_3_O_4_@OPTBA, Fe_3_O_4_@OPTBA@AuBP1, and Fe_3_O_4_@OPTBA@AuBP2, depicting the variations in residual mass of the samples with increasing temperature. TGA is an effective analysis tool to determine the organic contents of inorganic-organic composites. The organic composite should decompose completely when the temperature is high enough, while the inorganic composite should remain.

The thermograms exhibit a multiple-stage thermal decomposition. The first stage is in the 25–185 °C range and corresponds to the loss of moisture present in the sample (5%). The second stage is a bit more complex. It starts with the initial decomposition temperature (Ti) of the functionalized nanoparticles at 190.9 °C. The weight loss ranges from 190 to 540 °C and corresponds to the temperature of both the transformation from magnetite to hematite and the thermal decomposition of functionalization (both cross-linkers and peptides). There was no increase in weight from the oxidation of Fe_3_O_4_ to Fe_2_O_3_ because the TGA experiment was carried out under a nitrogen atmosphere. This indicated that the weight loss in the observed range is only due to the decomposition of the cross-linkers and peptides. As labeled in Fig. 5, the comparison of weight loss between Fe_3_O_4_@OPTBA, Fe_3_O_4_@OPTBA@ AuBP1, and Fe_3_O_4_@OPTBA@AuBP2 in the temperature range of 190–540 °C represents the quantity of peptide conjugation. Of the weight loss in Fe_3_O_4_@OPTBA, 24.60% correspond to the cross-linkers’ weight. Of the weight loss in Fe_3_O_4_@OPTBA@ AuBP1 and Fe_3_O_4_@OPTBA@AuBP2, 28.34% and 32.96% correspond to both cross-linkers and peptides, respectively. Upon calculation, AuBP2 had 4.62% more weight conjugated than AuBP1. The third stage is in the range of 680–730 °C and corresponds to the thermal decomposition of silane, namely, Si(OC_2_H_5_)_4_ → SiO_2_ + 2 O(C_2_H_5_)_2_.

### Quantification of peptide surface functionalization

In order to achieve the best gold-binding efficiency, each step of functionalization is optimized and precisely controlled. The measurement of chemical density on particle surface, called “parking area,” is utilized to describe the quality of each functionality. It refers to the average area in square nanometer occupied by each functional group. For the covalent coupling of peptides or proteins, the high-density surface would provide more reactive sites for conjugation, which effectively cover the underlying particle surface with charges. This helps to maintain particle suspension through charge repulsion and prevents non-specific binding.

For example, the silane functionalization of NH_2_/COOH was quantified by either colorimetric assay of amine density utilizing 4-NBA (Xiang et al. 2012) or Ni^2+^ titration together with PV for carboxyl density (Hennig et al. 2011). As a result, the amine density was quantified as 1.04 NH_2_/nm^2^ (3 × 10^−5^ mol/g), while the carboxyl density was 25 COOH/nm^2^ (8 × 10^−4^ mol/g). There are two possible explanations for the higher value in the carboxyl functionalization: (1) both carboxyl (COOH) and amide (CONH) groups presented in OPTBA were interacting with Ni^2^; under the assumption that both carboxyl and amide groups have the same surface binding stoichiometry factor (2.65) to Ni^2+^, the recalculated carboxyl density should be 12.5 COOH/nm^2^ (4 × 10^−4^ mol/g) and (2) the silane hydrolysis reaction is more efficient for carboxyl functionalization versus amine functionalization under the same reaction conditions. Regarding to a survey cited in Greg T. Hermanson’s “Bioconjugate techniques” (Hermanson 2013), carboxylate particles from different manufacturers have the average carboxylate parking area vary widely from about 1 to over 12.5 nm^2^. Here, that our carboxylate particles have the average parking area of 12.5/nm^2^ is seen as one of the excellent quality.

**Fig. 6:**
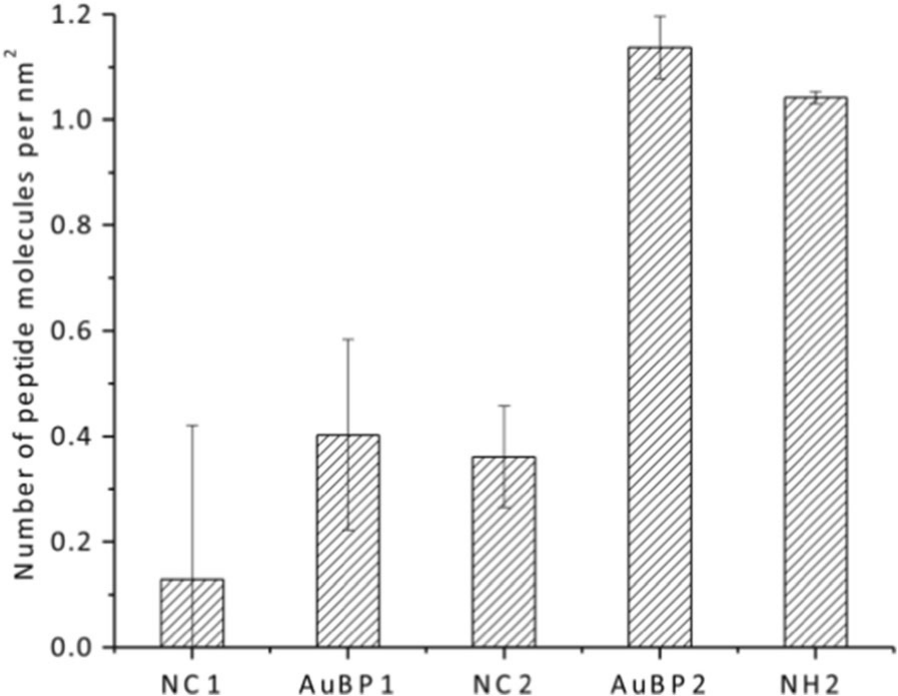
The peptide surface-grafting density of AuBP1 and AuBP2 with negative controls compared to amine functionalization

The resulting peptide conjugation is also optimized to reach the maximum value and quantified by monitoring the absorbance of the peptide at 280 nm in the solution, both before and after the reaction. The difference in absorbance intensity revealed the amount of AuBP grafted on the nanoparticles. However, the reduction of AuBP was not only caused by the covalent bond formation intended but also led by inevitable non-specific interactions, possibly induced by physical interactions like electrostatic forces, van der Waals forces, and hydrophobic effects. To distinguish these two interactions, Fe_3_O_4_@OTPBA NPs with non-activated carboxyl groups were selected as the negative control, under the assumption that no coupling reaction could happen between the NPs and peptides, since the reduction of peptides in the supernatant is caused only by non-specific interactions. The reaction was repeated three times for each peptide with the negative control. The result was converted to show the number of peptides per surface area and summarized in Fig. 6. AuBP1 has a surface density of 0.40 ± 0.18 molecules/nm^2^, with 0.13 ± 0.29 molecules/nm^2^ through non-specific interaction, while AuBP2 has a surface density of 1.14 ± 0.06 molecules/nm^2^, with 0.36 ± 0.09 molecules/nm^2^ through non-specific interaction. This data is comparable to the surface density of the amine functional group, which is quantified as 1.04 ± 0.01 molecules/nm^2^. The peptide conjugation reaction is favorable for AuBP2, likely because AuBP2 is more positively charged and more hydrophilic than AuBP1.

### Peptide surface functionalization

#### Gold-binding test

The interaction between Fe_3_O_4_@OTPBA@AuBP and gold was studied by three different sets of experiments. Fe_3_O_4_@OTPBA@AuBP was applied to a gold nanoparticle colloid solution and monitored by spectroscopy, Fe_3_O_4_@OTPBA@AuBP was applied to a gold chip for SPR measurement, and Fe_3_O_4_@OTPBA@AuBP was applied to a gold micropowder for a mining capacity test.

In the first method, Fe_3_O_4_@OTPBA@AuBP was applied to a gold nanoparticle colloid solution and the color change was recorded by UV-Vis spectroscopy to provide a rough description of the interaction. The second method used a SPR system to flow the Fe_3_O_4_@OTPBA@AuBP nanoparticle solution over the surface of gold chip. The last method used gold microparticles (40 μm) for the real capacity test.

#### Gold nanoparticle interaction

**Fig. 7:**
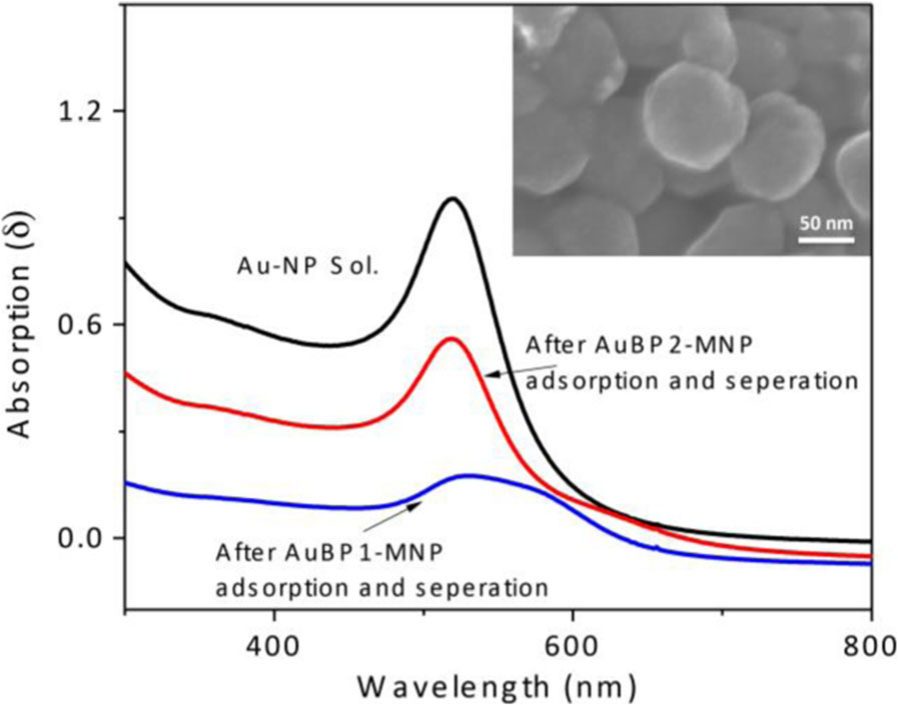
UV-Vis spectra of gold nanoparticle colloid before and after binding test with AuBP-MNPs. *Insert picture* represents the SEM picture of the AuBP-MNPs surface fully covered with Au NPs

Figure 7 shows the UV-Vis spectra of the gold nanoparticle colloid solution before and after the interaction with AuBP-MNPs. Since the gold nanoparticle solution has a surface plasmon resonance at 520 nm that is proportional to its concentration, the UV-Vis spectra obtained from the adsorption test could tell if the AuBP-MNPs is interacting. The results show that both AuBP1-MNPs and AuBP2-MNPs induced a decreased concentration of gold nanoparticle colloid. Interestingly, the gold nanoparticle solution showed not only a decreased absorption but also an induced aggregation after being treated with AuBP1-MNPs, which might be because of its hydrophobicity. The SEM picture also shows that the surface of the AuBP-MNPs was fully occupied by gold nanoparticles.

#### SPR measurement

The binding of Fe_3_O_4_@SiO_2_, Fe_3_O_4_@OTPBA@AuBP1, and Fe_3_O_4_@OTPBA@AuBP2 was monitored with a surface-based detection system, SPR spectroscopy. The calculated adsorption rate (*k*_*a*_), desorption rate (*k*_*d*_), equilibrium coefficient (*K*_eq_), and Gibbs free energy of adsorption for Fe_3_O_4_@OTPBA@AuBP1 and Fe_3_O_4_@OTPBA@AuBP2 were summarized in Table 2. Each nanoparticle was tested for adsorption onto the gold surface in various concentrations, from 20 to 696 pM (Fig. S2). Each nanoparticle system showed different adsorption behaviors at a given concentration (Fig. S3). Both Fe_3_O_4_@OTPBA@AuBP1 and Fe_3_O_4_@OTPBA@AuBP2 fit to the Langmuir adsorption model, while bare NPs did not. The peptides, AuBP1 and AuBP2, had been reported to exhibit Langmuir adsorption kinetics on a gold surface (Hnilova et al. 2008). The experiments shown here indicated that these peptides could still be adsorbed to the gold surface with comparable kinetic constants even when coated on the NP surface. The AuBP-MNPs exhibit almost 10^2^-fold higher adsorption and desorption kinetics compared to the peptide alone (Hnilova et al. 2008). This increase might be due to a combination of reasons: (1) the high refractive index of magnetic nanoparticles, which will give a higher magnitude of plasmonic shift, allows more sensitive detection of binding affinity; (2) the high molecular weight and size of the nanoparticles compared to the free peptide allows to overcome possible mass transport limitations and calculation of real *K*_*a*_; and (3) multivalent interactions between the nanoparticles (compared to the case of peptide only) and the surface increase the binding affinity.

**Table 2:** Adsorption rate (*k*_*a*_), desorption rate (*k*_*d*_), equilibrium coefficient (*K*_eq_), and Gibbs free energy of adsorption for Fe_3_O_4_@OTPBA@AuBP1 and Fe_3_O_4_@OTPBA@AuBP2

	*k*_*a*_ (× 10^7^ M^−1^ s^−1^)	*k*_*d*_ (× 10^−2^ s^−1^)	*K*_eq_ (× 10^8^ M^−1^)	Δ*G* (kcal/mol)
Fe_3_O_4_@OTPBA@AuBP1	7.73 ± 2.44	2.87 ± 0.8	26.934 ± 11.34	−12.7351 ± 0.2
Fe_3_O_4_@OTPBA@AuBP2	2.46 ± 4.46	7.24 ± 2.6	3.398 ± 6.28	−11.5209 ± 0.6

The values of *k*_*a*_ and *k*_*d*_ are given within 95% confidence intervals, while the errors in *K*_eq_ and Δ*G* are determined by the propagation of error in the *k*_*a*_ and *k*_*d*_ values (see the MATLAB code for details)

When the binding behaviors of AuBP1 and AuBP2-coated NPs were compared, AuBP1-MNP showed a higher adsorption and a lower desorption than AuBP2-MNP, which in return resulted in a higher equilibrium constant of more than 8-fold. This difference could be also followed in the peptide-alone case with a very slight difference between two peptides (Hnilova et al. 2008). Even though both systems were self-driven in terms of free energy calculations, the binding energy of AuBP1-MNP was ~1.2 kcal/mol lower than the binding energy of AuBP2-MNP.

#### Gold micropowder mining test (including pH and temperature effects)

A bench-top mining test was performed by using 1 mg of nanoparticle to adsorb gold from a solution made by floating 20 mg of gold micropowder into 5 mL Milli-Q water. Here, a maximum binding capacity in an ideal working condition is shown rather than mimic the real mine tailings. This is due to the fact that the composition of tailings varies a lot depending on the resource being mined, processing technology used, and geology at the mine site. In order to utilize this technology into the real mine tailings, a pretreatment that bring the tailing sample into a demanding concentration and size is in need. As a result, a high degree of gold adsorption onto the peptide-functionalized surfaces was observed in the aqueous solution. ICP-MS was used to measure the concentration of the iron oxide nanoparticles, the mined gold, and the leftover gold. At this concentration, 1 mg of nanoparticle could adsorb ~10 mg, 74% of the total amount of gold in the solution and separate them from the solution (pH = 7). Interestingly, the removal (Au absorption onto MNP) efficiency is influenced strongly by the pH of the mining process but not its temperature. A relatively high removal efficiency was observed at pH = 2 (~15 mg, 95%), in the contrast to observations at pH = 10 (~9 mg, 61%). This indicates the effect of peptide charge on inorganic binding efficiency, and for this particular system, it provides a tunable recovery during mining applications. When the mining test was performed at three different temperatures (room temperature, 65 and 95 °C) at pH = 7, a consistent 74~75% gold was removed from the solution. This indicates the stability of peptides on the MNP surface and suggests possible utilization of MNPs for mining applications that require high temperatures. It is also significant to note that a 2~3% nanoparticle weight loss was observed for all the tests. This strongly suggests that a more effective recycle system need to be adapted when apply to a large-scale mining process.

## Conclusions

Gold-binding peptide-functionalized, magnetic nanoparticles were prepared by a three-step protocol under aqueous reaction conditions at room temperature. Highly uniform quasi-spherical iron oxide magnetic nanoparticles were synthesized using a hydrothermal reaction. Then, amine or carboxylate groups were introduced onto the surface of the magnetic nanoparticle. Finally, gold-binding peptides (AuBP1 and AuBP2) were conjugated to the functionalized surface via conjugation reaction. The structure, morphology, and magnetism of the AuBP-MNP were thoroughly investigated. Surface coverage of the peptides was quantified as 0.40 ± 0.18 molecules/nm^2^ for AuBP1-MNP and 1.14 ± 0.06 molecules/nm^2^ for AuBP2-MNP. Applying AuBP-MNPs to the Au nanoparticle solution (1–3 nm) resulted in a dramatic reduction of Au nanoparticle concentration via magnetic separation. The SPR experiment revealed that the interaction between AuBP-MNPs and the Au surface fits the Langmuir adsorption model. The AuBP-MNPs exhibit almost 10^2^-fold higher adsorption and desorption kinetics than peptides alone. Compared to AuBP2-MNP, AuBP1-MNP showed a higher adsorption and a lower desorption, which resulted in an equilibrium constant more than 8-fold higher. Finally, the bench-top mining test with gold microparticles demonstrated that 1 mg AuBP-MNPs could sufficiently recover ~10 mg of gold powder with 2~3% nanoparticle weight lost. The mining performance of AuBP-MNPs is highly sensitive to pH but not temperature. Future work focuses on generalization of a systematic recycle strategy for the recovery of these magnetic nanoparticles, including changing pH and ionic strength and using competitive binding.

## Electronic supplementary material

ESM 1 (DOCX 569 kb)
