# Supplementary material for: Peptide-functionalized iron oxide magnetic nanoparticle for gold mining
Source: J Nanopart Res. 2017 Feb 17;19(2):74. doi: 10.1007/s11051-017-3752-7 (PMC5315719; doi:10.1007/s11051-017-3752-7)
Supplement: Supplementary file 1 — (DOCX 569 kb) [file 11051_2017_3752_MOESM1_ESM.docx]

**Peptide Functionalized Iron Oxide Magnetic Nanoparticle for Gold Mining**

**Wei-Zheng Shen,^†,♯^Sibel Cetinel,^†,♯^ Kumakshi Sharma,^†,♯^ Elham Rafie Borujeny,^†,♯^ Carlo Montemagno^†,♯,^**^‡,^*****

^†^Ingenuity Lab, 1-070C, 11421 Saskatchewan Drive NW, T6G 2M9, Edmonton AB, Canada;

^♯^Departement of Chemical and Materials Engineering, University of Alberta, T6G 2V4, Edmonton, AB, Canada;

^‡^National Institute of Nanotechnology, 11421 Saskatchewan Drive NW, T6G 2M9, Edmonton AB, Canada;

*Corresponding Author: Carlo Montemagno. E-mail: montemag@ualberta.ca

Supporting Information


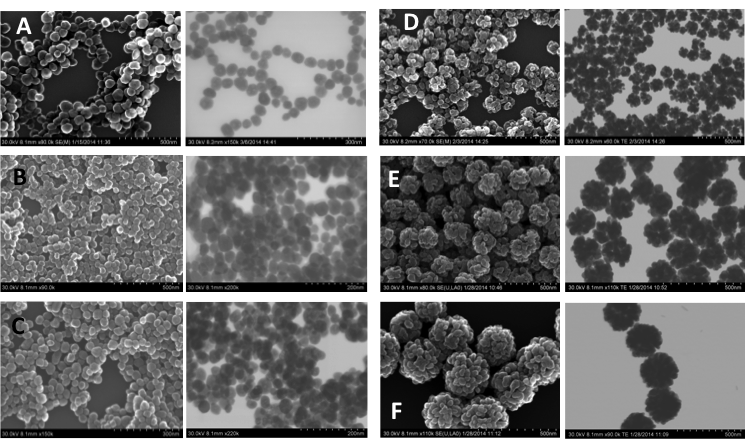


**Fig. S1** SEM of nanoparticles with uniform sizes of 70 nm (A), 50 nm (B), and 40 nm (C); using higher concentration of precursor and longer reaction time, larger nanoclusters with uniform sizes of 150 nm (D), 180 nm (E) and 250 nm (F) could be obtained.

**A**


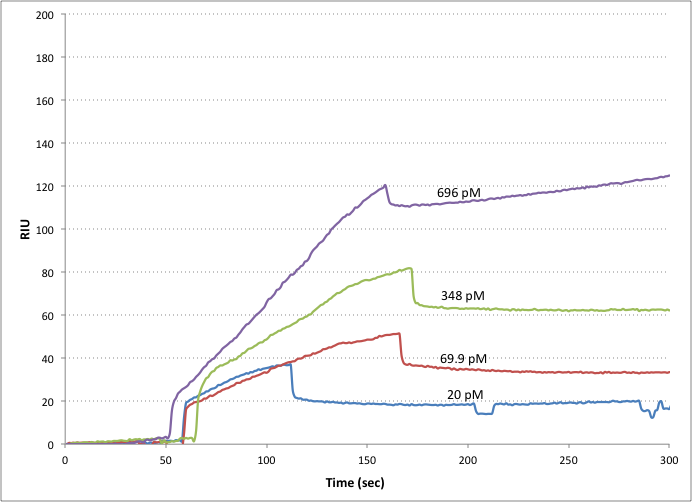


**B**

C

**Fig. S2** SPR sensogram of Fe_3_O_4_@SiO_2_, Fe_3_O_4_@OPTBA@AuBP1 and Fe_3_O_4_@OPTBA@AuBP2 with concentration titration.

**Fig. S3** SPR sensogram of Fe_3_O_4_@SiO_2_, Fe_3_O_4_@OPTBA@AuBP1 and Fe_3_O_4_@OPTBA@AuBP2, each at 69.6 pM concentration.

**Calculation of the concentration of the nanoparticles used for the SPR experiments**

The average diameter of nanoparticles is 70 nm.

Volume of one nanoparticle:

V= (4/3)πr^3^ = (4/3)×3.14×35^3^ nm^3^ = 1.7×10^5^ nm^3^ = 1.7×10^-16^ cm^3^

Weight of one nanoparticle:

W= dV = 5 g/cm^3^ × 1.7×10^-16^ cm^3^ = 8.5×10^-16^ g

Surface area of one nanoparticle:

S = 4πr^2^ = 4×3.14×35^2^ nm^2^ = 1.5×10^4^ nm^2^

Therefore, 1mg/mL of nanoparticle is equal to nanoparticle number concentration of

1mg/mL = 1mg/8.5×10^-16^g/mL = (1mg/8.5×10^-13^ mg)/mL = 1.17647×10^12^ NPs/mL

Number concentration converts to molar concentration

1.2×10^12^ NPs/mL = (1.17647×10^12^ NPs/mL)/(6.02×10^23^) = 1.96 ×10^-12^ mol/mL =1.96 ×10^3^ pM

To calculate the peptide-functionalized nanoparticle, peptide surface density is average 1peptide/nm^2^

Weight of one peptide functionalized nanoparticle:

W = W_NP_ + W_peptide_ = 8.5×10^-16^ g + 1454.74×(1.5×10^4^)/(6.02×10^23^) = 8.863685×10^-16^ g

Therefore, 1mg/mL of peptide-functionalized nanoparticle is equal to number concentration of

1mg/mL = 1mg/8.863685×10^-16^g/mL = 1.12820 ×10^12^ NPs/mL

Number concentration converts to molar concentration

1.12820 ×10^12^ NPs/mL = (1.12820×10^12^ NPs/mL)/(6.02×10^23^) = 1.87×10^-12^ mol/mL =1.87 ×10^3^ pM
